# Icariin Ameliorates Diabetic Renal Tubulointerstitial Fibrosis by Restoring Autophagy *via* Regulation of the miR-192-5p/GLP-1R Pathway

**DOI:** 10.3389/fphar.2021.720387

**Published:** 2021-07-19

**Authors:** Zhirong Jia, Kaiwei Wang, Yameng Zhang, Yalei Duan, Kang Xiao, Shuo Liu, Xuansheng Ding

**Affiliations:** ^1^ School of Basic Medicine and Clinical Pharmacy, China Pharmaceutical University, Nanjing, China; ^2^ Precision Medicine Laboratory, School of Basic Medicine and Clinical Pharmacy, China Pharmaceutical University, Nanjing, China

**Keywords:** diabetic kidney disease, diabetic tubulointerstitial fibrosis, autophagy, icariin, testosterone, miR-192-5p, glucagon-like peptide-1 receptor

## Abstract

Tubulointerstitial fibrosis is one of the most common pathological features of diabetic nephropathy. Autophagy, an intracellular mechanism to remove damaged or dysfunctional cell parts and maintain metabolic homeostasis, is inhibited in diabetic neuropathy. Icariin is a traditional Chinese medicine extract known for nourishing the kidney and reinforcing Yang. In this study, we investigated the effects and mechanism of Icariin on renal function, autophagy, and fibrosis in type 2 diabetic nephropathic rats and in high-glucose-incubated human renal tubular epithelial cells and rat renal fibroblasts (*in vitro*). Icariin improved diabetes, renal function, restored autophagy, and alleviated fibrosis in type 2 diabetic neuropathic rats and *in vitro*. After we applied autophagy-related gene 5-small interfering RNA, we found that fibrosis improvement by Icariin was related to autophagy restoration. By detecting serum sex hormone levels, and using dihydrotestosterone, siRNA for androgen receptor, and the androgen receptor antagonist Apalutamide (ARN-509), we found that Icariin had an androgen-like effect and restored autophagy and reduced fibrosis by regulating the androgen receptor. In addition, miR-192-5p levels were increased under high glucose but reduced after dihydrotestosterone and Icariin treatment. Furthermore, dihydrotestosterone and Icariin inhibited miR-192-5p overexpression-induced fibrosis production and autophagy limitation. Glucagon-like peptide-1 receptor (GLP-1R) was downregulated by high glucose and overexpression of miR-192-5p and could be restored by dihydrotestosterone and Icariin. By using ARN-509, we found that Icariin increased GLP-1R expression by regulating the androgen receptor. GLP-1R-siRNA transfection weakened the effects of Icariin on autophagy and fibrosis. These findings indicate that Icariin alleviates tubulointerstitial fibrosis by restoring autophagy through the miR-192-5p/GLP-1R pathway and is a novel therapeutic option for diabetic fibrosis.

## Introduction

Millions of people are diagnosed with diabetes or prediabetes every year. Diabetes is one of the fastest growing diseases in the world and is expected to affect 693 million adults by 2045 (Cole and Florez, 2020). With the increase in the prevalence of diabetes mellitus, diabetic nephropathy (DN) is now the leading cause of chronic kidney disease worldwide and the primary cause of end-stage renal disease (ESRD) in several countries (Thomas et al., 2015). Unfortunately, effective treatments for DN are lacking, and the development of effective treatments to block DN progression is urgent.

Diabetic neuropathy can occur in patients with either type 1 diabetes mellitus (T1DM) or type 2 diabetes mellitus (T2DM) (Molitch et al., 2015). Although the prevalence of both T1DM and T2DM is increasing, T2DM accounts for approximately 95% of all diabetic individuals, and the most pronounced increase in DN is seen among individuals with T2DM (Flyvbjerg, 2017). In recent years, anti-T2DM effects of some kinds of flavonoids have been reported (Xu et al., 2018). Icariin (ICA) is a major constituent of a flavonoid isolated from the plant *Herba epimedii*. A wide range of pharmacological effects has been attributed to ICA, including: improvement in neuronal insulin resistance Zou et al. (2020) and testicular dysfunction (Zhao et al., 2020); protection of hippocampal neurons Liu et al. (2019) and nucleus pulposus cells (Hua et al., 2020); potential against Alzheimer's Disease (Angeloni et al., 2019); and notably, as a traditional Chinese medicine, nourishment of the kidney and reinforcement of Yang. Recent research has demonstrated that ICA treatment reduces blood glucose levels in type 2 diabetic rats and protects pancreatic function Han et al. (2015), Li et al. (2020), Zhang and Qiu (2020), suggesting that ICA has an anti-T2DM effect. However, the exact mechanism of ICA on T2DN is yet unknown. One of the most common characteristics of DN is tubulointerstitial fibrosis, which is caused by excess accumulation of extracellular matrix (ECM), and accelerates renal failure and appears early in diabetic kidney injury (Liu, 2011). Autophagy is deficient or insufficient in DN and the restoration of autophagy may be a novel therapeutic option (Warren et al., 2019; Zhao et al., 2019). Research has shown that autophagy has obvious significance in ameliorating tubulointerstitial fibrosis (Nam et al., 2019). However, the effect and mechanism of ICA regulating tubulointerstitial fibrosis and autophagy is still unclear.

The mechanism of type 2 diabetes in males is related with the reduced testosterone levels. Compared with the normal men, male patients with type 2 diabetes have decreased testosterone levels (Espeland et al., 2015; Hylmarova et al., 2020). Notably, combined inhibition of aromatase activity and dihydrotestosterone supplementation attenuates renal injury in male diabetic rats (Manigrasso et al., 2012). Epimedii Herba is an herbal medicine originating from several plants of the genus *Epimedium*. It is a major therapeutic option for kidney Yang deficiency syndrome, which is closely related to androgen hormones (Cho et al., 2017). Icariin, as the main hydrophilic ingredient of Epimedium brevicornu Maxim, has been proven to be a plant sex hormone Liu et al. (2021), and has testosterone mimetic properties (Zhang and Yang, 2006). However, how testosterone improves diabetic renal injury in males is largely unclear and whether ICA attenuates renal injury and fibrosis by the same mechanism as testosterone deserves investigation.

GLP-1R (Glucagon-like peptide-1 receptor), which is present in various organs, such as the liver, brain and kidney, is an important pharmacological target for T2DM (Aroda, 2018). A recent study showed that GLP-1R plays an important role in regulating non-diabetic and diabetic renal fibrosis Li et al. (2018), Yu et al. (2020), and *Glp1r* mRNA levels positively correlate with serum testosterone concentrations in normal male mice and in diabetic models (Zhu et al., 2019). Research has demonstrated that miR-192 induces fibrosis in human renal tubular epithelial cells by targeting GLP-1R (Jia et al., 2018). The miR-192 was a potential target for the treatment of diabetic nephropathy. The specific reduction of renal miR-192 decreases renal fibrosis and improves proteinuria (Putta et al., 2012). We speculate that ICA and testosterone may regulate miR-192/GLP-1R pathway. In this study, we investigated whether ICA regulates autophagy and tubulointerstitial fibrosis by modulating the miR-192/GLP-1R pathway.

## Materials and Methods

### The Effect of ICA on STZ-Induced Type 2 Diabetic Kidney Disease

Six-week-old male Sprague-Dawley rats weighing between 160 and 180 g (*n* = 50) were purchased from Zhejiang Experimental Animal Center (Hangzhou China). All experimental rats were maintained in environmentally controlled animal facilities at China Pharmaceutical University. All animal procedures were approved by the China Pharmaceutical University Experimental Animal Ethics Committee. After 1 week of acclimatization to the environment, the mice were randomly divided into two groups: a normal control group (*n* = 8), and a diabetic model group (*n* = 42). Rats in the normal control group were fed a standard diet (10% calories from fat) and the other rats were fed a high-sugar and high-fat diet (ingredients: 10% refined lard, 20% sucrose, 2% cholesterol, 1% sodium cholate, and 67% common food; 68% calories from fat and sugar, 50% calories from fat) Guo et al. (2012) for 4 weeks. High sugar and high fat-fed rats were injected intraperitoneally with a single dose of streptozotocin (STZ) (35 mg/kg; Sigma, St. Louis, MO, United States) dissolved in freshly prepared 0.1 M citrate buffer (pH 4.2), while the normal control rats received an equal volume of citrate buffer. Random blood glucose (RBG) levels and the fasting blood glucose (FBG) levels were detected 3 days and 1 week, respectively, after STZ injection. The rats with RBG levels ≥16.7 mM and FBG levels ≥11.1 mM were considered to be diabetic (*n* = 38). Blood glucose meter and blood glucose test strips were obtained from Roche (Basel, Switzerland). Diabetic rats were randomly chosen to receive treatment with ICA (Yangtze River Pharm Co., Ltd. Taizhou, Jiangsu, China) by gavage (20, 40, and 80 mg/kg/d) as the DN + ICA group (*n* = 9–10 per group). Other DN rats (*n* = 10) and NC rats (*n* = 8) were administered an equal volume of saline. After a prolonged time, when the blood creatinine and blood urea nitrogen in the model group were significantly higher than those in the normal group and the creatinine clearance rate was significantly lower than the normal group, the rats were considered to have DN. After 12 weeks of administration, the metabolic cages were used to collect 24 h urine from each rat. The volumes for the 24 h urine samples were recorded, and 10 ml aliquots were centrifuged at 3,500 rpm for 10 min. Levels of urine protein and creatinine were immediately detected in the collected supernatant, and the remaining urine samples were stored at −80°C. Finally, the rats were sacrificed, and their kidney tissues were obtained. For hematoxylin-eosin (H&E), Masson staining, and IHC, part of kidney tissues were fixed in 4% paraformaldehyde. The remaining kidney tissues were preserved at −80°C for Western blot analysis. The blood samples of all the rats were centrifuged and the serum was collected and stored at −80°C for the detection of certain biochemical indexes.

### ELISA Assay

The levels of blood urea nitrogen, serum creatinine, urinary creatinine and urine protein were detected by ELISA assay (Nanjing JianCheng Bioengineering Institute, Nanjing, China). The ELISA kits detecting the levels of testosterone, LH, and FSH were purchased from Elabscience Biotechnology Co., Ltd (Wuhan, China). A rat insulin ELISA kit was obtained from Jiangsu Yutong Biotechnology Co., Ltd (Nanjing, China). All ELISA assays were performed according to the manufacturer’s instructions.

### Cell Culture and Treatment

Human renal tubular epithelial cells (HK-2) were obtained from FENGHUISHENGWU (Changsha, Hunan, China). Rat kidney fibroblasts (NRK-49F) were a gift from the Second Affiliated Hospital of Anhui Medical University. Cells were cultured in Dulbecco’s Modified Eagle’s medium (DMEM) containing 10% FBS (fetal bovine serum (ExCell Bio, Shanghai, China) and penicillin-streptomycin solution (1X, KeyGEN BioTECH, Nanjing, China) in an atmosphere of 5% CO_2_ at 37°C. For the experimental study, HK-2 and NRK-49F cells were maintained in DMEM with normal glucose (5.6 mM glucose, NG). Cells were seeded at ∼60% confluence, then cultured in FBS-free DMEM for 24 h and subsequently exposed to DMEM-containing 30 mM glucose (HG) and ICA of different concentrations (Yangtze River Pharm Co., Ltd. Taizhou, Jiangsu, China), 50 and 100 nM DHT (Stanolone, Shanghai yuanye Bio-Technology Co., Ltd, Shanghai, China), 80 nM ARN-509 (MCE, Shanghai, China), 5 µM chloroquine (CQ) (from MCE), or 10 and 100 nM Rapamycin (MCE) for an additional 48 h.

### Western Blot Analysis

Proteins were extracted from the cells and tissues in lysis buffer (KeyGEN BioTECH, Nanjing, China) and protease inhibitor cocktail (KeyGEN) for Western blotting. The extracted proteins were separated by polyacrylamide SDS gels and electrophoretically transferred onto PVDF membranes (Millipore, MA, United States). The membranes were probed with the indicated antibodies overnight at 4°C. Antibodies used for Western blots were: anti-LC3 and mTOR (Cell Signaling Technology, Boston, MA, United States, 1: 2,500 dilution), anti-p62, ATG5, and *p*-mTOR (Bimake, Houston, TX, United States, 1: 3,000 dilution), anti-Collagen I and GLP-1R (Bioss, Beijing, China, 1: 1,000 dilution), anti-FN (Abcam, Cambridge, United Kingdom, 1: 2,000 dilution), anti-α-SMA (1: 2,000 dilution), AR (1: 1,000 dilution) and GAPDH (1: 10,000 dilution) (Proteintech, WUHAN SANYING, WuHan, China) antibodies. After washing, membranes were incubated at room temperature with the HRP-conjugated goat anti-rabbit IgG secondary antibody (Beyotime, Nantong, Jiangsu, China) for 1 h. The electrochemical luminescent substrates (Vazyme, Nanjing, China) were used according to the manufacturer’s protocol in order to visualize the proteins of interest using in the Tanon imaging system (Tanon, Shanghai, China). The relative expressions were quantified densitometrically using the ChemiScope analysis software and calculated according to the reference bands of GAPDH.

### Apoptosis Detection

An Annexin V-FITC apoptosis kit (Vazyme, Nanjing, China) was used to determine the number of apoptotic cells according to the manufacturer’s instructions. The NRK-49F cells were sorted using a FACS flow cytometer (Miltenyi Biotec). The total apoptotic rates were assessed and analyzed by flow cytometry after indicated treatment for 48 h and the results were analyzed using FlowJo VX software.

### Immunohistochemistry, HE and Masson Staining

The tissues were vacuum inflation-fixed and paraffin embedded, and 5-μm sections were cut. Sections were used for immunohistochemical examination. Immunohistochemical staining was performed with the following primary antibodies: rabbit monoclonal anti-LC3 (Cell Signaling Technology), rabbit recombinant monoclonal anti-p62 (Bimake), rabbit monoclonal anti-FN (Abcam), and rabbit polyclonal anti-GLP-1R (Bioss) followed by incubation with horseradish peroxidase (HRP)-conjugated goat anti-rabbit IgG secondary antibody according to the kit instructions (ZSGB-BIO, Beijing, China). Peroxidase conjugates were subsequently visualized by using diaminobenzidine (DAB) solution. The sections were then counterstained with hematoxylin and mounted on cover slips. Between each step, the cells were extensively rinsed three times for 5 min each time. HE and Masson staining were performed according to the manufacturer’s instructions. The staining kits were purchased from Nanjing JianCheng Bioengineering Institute (Nanjing, China). Staining was photographed using a Leica microscope (DM2500, Wetzlar, Hesse, Germany). For each rat, five fields were chosen to obtain the average of integrated optical density (iod) of IHC staining and the average collagen area indicated by Masson staining by Image Pro Plus (IPP) software. Average areas from 5 rats were determined, and the analysis was done blindly.

### Confocal Imaging

For the mCherry-GFP-LC3B assay, HK-2 cells were seeded in 6-well plates on microscope glass coverslips, infected with mCherry-GFP-LC3B adenovirus (Beyotime) for 24 h, and treated with NG or HG, CQ and ICA for another 48 h. Following treatment, cells were fixed with 4% paraformaldehyde in phosphate-buffered saline (PBS) and incubated with DAPI (1:1,000) for 10 min, then examined by confocal microscopy (CLSM, Carl Zeiss LSM800, Jena, Germany).

### Transfection With miR-192-5p Mimic or Inhibitor

HK-2 and NRK-49F cells were seeded in 6-well plates at a density of 1 × 10^5^ cells/mL. At 70% confluence, the cells were transfected with 50 nM micrOFF miR-192-5p or the inhibitor control, or with 30 nM micrON miR-192-5p (miR-192-5p mimic) or the mimic control (RiboBio, Guangzhou, China) using Lipofectamine 2000 (Life Technologies Corporation, Gaithersburg, MD, United States), according to the manufacturer’s instructions. The RNA-lipid complexes were added to the cells, and the medium was replaced after 6 h. After the cells were transfected for 48 h, they were treated with HG, ICA, or DHT for 48 h. The mature sequences of miR-192-5p in human and rat were same and the sequences were 5′-CUG​ACC​UAU​GAA​UUG​ACA​GCC-3′.

### Quantitative Real-Time PCR

Total RNA was prepared from cells using TRIzol reagent (Ambion, Austin, TX, United States) according to the manufacturer’s instructions. RNA quality was measured using a NanoDrop ND-1000 spectrophotometer (Thermo Fisher Scientific, Waltham, MA, United States). Approximately 500 ng of RNA was used for cDNA synthesis with a HiScript III RT SuperMix for qPCR kit (Vazyme, Nanjing, China). ChamQ SYBR qPCR Master Mix Kit (Vazyme) was used to quantify mRNA expression, and GAPDH was used as an internal control. The mRNA primers are described in Table 1. For the specific detection of miR-192-5p expression, the stem-loop real-time PCR was performed as previously described (Chen et al., 2005). Approximately 1 μg of RNA was used for cDNA synthesis with a miRNA 1st Strand cDNA Synthesis Kit (by stem-loop) (Vazyme). The miRNA Universal SYBR qPCR Master Mix (Vazyme) was used to quantify miR-192-5p expression, and RNU6 was used as an internal control. The miRNA primers for reverse transcription and quantification are described in Table 2. PCR reactions were conducted on ABI StepOne PCR system (Applied Biosystems, Foster City, CA, United States) and results were analyzed with the ΔΔCt method.

**TABLE 1:** mRNA primer sequences for PCR.

Gene	Forward primer sequence (5′-3′)	Reverse primer sequence (5′-3′)
Human COL1A1	AAC​TGG​TAC​ATC​AGC​AAG​AA	CCA​TAC​TCG​AAC​TGG​AAT​CC
Human ACTA2	TGG​CTA​TTC​CTT​CGT​TAC​TA	AAG​TCC​AGA​GCT​ACA​TAA​CA
Human FN1	GTC​ACA​GAG​GCT​ACT​ATT​ACT	GCT​CGC​TCT​TCT​GAT​TAT​TC
Human SQSTM1	TGG​AGT​CGG​ATA​ACT​GTT​C	CGGATTCTGGCATCTGTA
Human BECN1	CGT​GGA​ATG​GAA​TGA​GAT​T	GTA​AGG​AAC​AAG​TCG​GTA​TC
Human GAPDH	CTT​CTT​TTG​CGT​CGC​CAG​CCG​A	ACC​AGG​CGC​CCA​ATA​CGA​CCA​A

**TABLE 2:** miRNA primer sequences for PCR.

Gene	Forward primer sequence (5′-3′)	Reverse primer sequence (5′-3′)
miR-192-5p	GCG​CGC​TGA​CCT​ATG​AAT​TG	AGT​GCA​GGG​TCC​GAG​GTA​TT (Universal)
miR-192-5p-RT	GTC​GTA​TCC​AGT​GCA​GGG​TCC​GAG​GTA​TTC​GCA​CTG​GAT​ACG​ACG​GCT​GT	—
RNU6	CTTCGGCAGCACATATAC	GAATTTGCGTGTCATCCT
RNU6-RT	GAATTTGCGTGTCATCCT	—

### Cell Viability Assays

The cell viability assay was performed as previously described (Yao et al., 2010). Briefly, 5 × 10^3^ cells/well were seeded in 96-well plates, leaving six wells empty for background measurements; the outermost wells were filled with sterile PBS. Twenty-four hours after seeding, the NKR-49F cells were treated with HG and ICA or transfected with miR-192-5p mimic. Cell number and viability were detected using the CCK-8 kit (Cell Counting Kit-8, APExBIO, Houston, United States) according to manufacturer’s instructions by measuring the absorbance at 450 nm at indicated time points. Cell viability was then calculated from the absorbance values as a percentage of the control cells after removing the mean background values.

### Transfection of SiATG5, siAR, and siGLP-1R

In all experiments, 150 pmol siRNA (the target sequences are listed in Table 3) (Genomeditech, Shanghai, China) were used to transfect 70–80% confluent cells according to the manufacturer's instructions. The Lipofectamine 2000 reagent (Life Technologies, Carlsbad, CA, United States) was used to deliver siRNA into HK-2 cells growing in serum‐free opti‐MEM media (Gibco, Gaithersburg, MD, United States). After 6 h, the medium containing the siRNA‐lipid complexes was replaced with DMEM containing 10% FBS. And subsequent experiments were completed at indicated times after transfection.

**TABLE 3:** Specific sequences for siRNA.

Gene	Sense (5′-3′)	anti-sense (5′-3′)
Human ATG5	CAC​UAG​GAG​AUC​UCC​UCA​A tt	UUG​AGG​AGA​UCU​CCU​AGU​G tt
Human AR	UCC​CCA​AGC​CCA​UCG​UAG​A tt	UCU​ACG​AUG​GGC​UUG​GGG​A tt
Human GLP-1R	UCU​GCA​UCG​UGG​UAU​CCA​A tt	UUG​GAU​ACC​ACG​AUG​CAG​A tt

### Statistical Analysis

Data are expressed as mean values ± Standard Deviation. Unpaired Student’s t-test was used when comparing two groups. One-way ANOVA with Dunnett’s test was used to compare multiple groups. Statistical analysis was performed using Prism 8.00 software (GraphPad, San Diego, CA, United States). The differences were considered significant for *p* < 0.05.

## Results

### ICA Ameliorates Diabetes in T2DN Rats

The rat T2DN model was established, and ICA was administrated as indicated in the Methods section. After 4 weeks of high fat-high sugar diet, the body weight and the mean growth rates of the diabetic model group was significantly higher than that of the standard chow control group. However, after STZ injection, the body weight and the mean growth rates of model group began to decline gradually, and after 3 weeks of STZ injection, the average body weight of the model group was reduced significantly relative to the normal group (Supplementary Figure 1A–B). Within 4 weeks of high fat-high sugar diet, there was no significant difference in calories intake per 100 g body weight between the normal group and the model group. After STZ injection, the calories intake per 100 g body weight of the model rats increased steadily. After 2 weeks of STZ injection, the calories intake per 100 g body weight of the model group increased significantly compared to the control group, indicating that the calories intake of the rats in the early stage of diabetes began to increase (Supplementary Figure 1C). Within 4 weeks of high fat-high sugar diet, due to the significant increase in body weight of the model group, the water intake per 100 g body weight decreased compared with the normal group. After STZ injection, the water intake of the normal and model group showed a similar trend as calories intake (Supplementary Figure 1D). Then the effects of ICA on body weight, calories intake, and water intake were investigated. The results showed that compared to the normal group, the body weight and the mean growth rates of model group was decreased, and the calories intake and water intake was significantly increased. After 12 weeks of ICA administration, ICA improved the body weight, the mean growth rates and significantly decreased calories intake and water intake relative to those untreated in the model group (Supplementary Figure 2A–D). In addition, compared with the normal group, the RBG, FBG, and FISN (fasting insulin) levels were increased significantly (Supplementary Figure 3A–D, Supplementary Figure 4A, B), and insulin sensitivity index (ISI) was decreased (Supplementary Figure 4C) in the model group after STZ injection. After 12 weeks of ICA administration, the levels of RBG, FBG, and FISN were significantly decreased (Supplementary Figure 3E–F, Supplementary Figure 4D), and the ISI was obviously increased (Supplementary Figure 4E) in ICA-treated group compared to the model group. These results indicate that ICA attenuates diabetes in the T2DN model.

### ICA Ameliorates Renal Function in T2DN Rats

**FIGURE 1:**
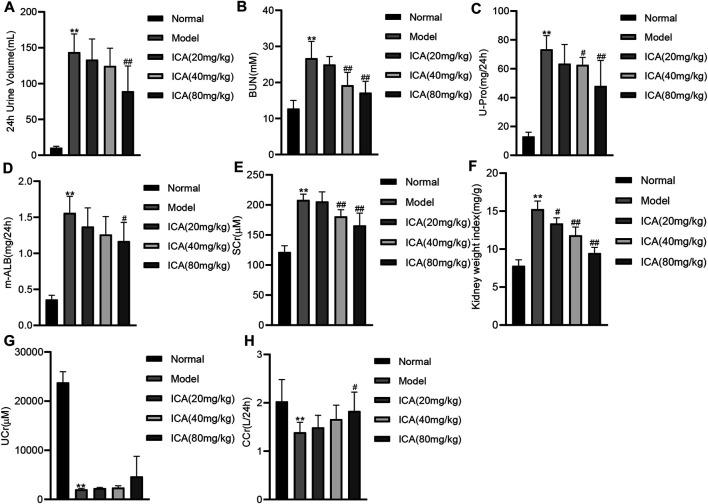
ICA improves renal function in T2DN rats **(A)** The 24 h urinary volume **(B)** Blood urea nitrogen (BUN) **(C)** 24 h urinary protein **(D)** 24 h urine microalbumin **(E)** Serum creatinine (Src) **(F)** The ratio of kidney weight to body weight **(G)** Urinary creatinine **(H)** Creatinine clearance were measured by ELISA. Data are expressed as mean ± SD (*n* = 8). **p < 0.01, *vs* the Normal group; ^#^ *p* < 0.05, ^##^ *p* < 0.01, vs the Model group.

The 24 h urinary volume (Figure 1A), blood urea nitrogen (BUN) (Figure 1B), 24 h urine protein (U-Pro) (Figure 1C), 24 h urine microalbumin (m-ALB) (Figure 1D), serum creatinine (SCr) (Figure 1E), and the ratio of kidney weight to body weight (kidney weight index) (Figure 1F) were all significantly higher in the model group. However, these values decreased dose-dependently in ICA-treated groups. The urinary creatinine (UCr) (Figure 1G) and creatinine clearance (CCr) (Figure 1H) were significantly decreased in the diabetic model group compared to the normal group, and ICA improved the UCr and CCr in a dose-dependent manner. These results suggest that ICA improves renal function in T2DN rats.

### ICA Induces Autophagy and Reduces Renal Fibrosis in T2DN Rats

**FIGURE 2:**
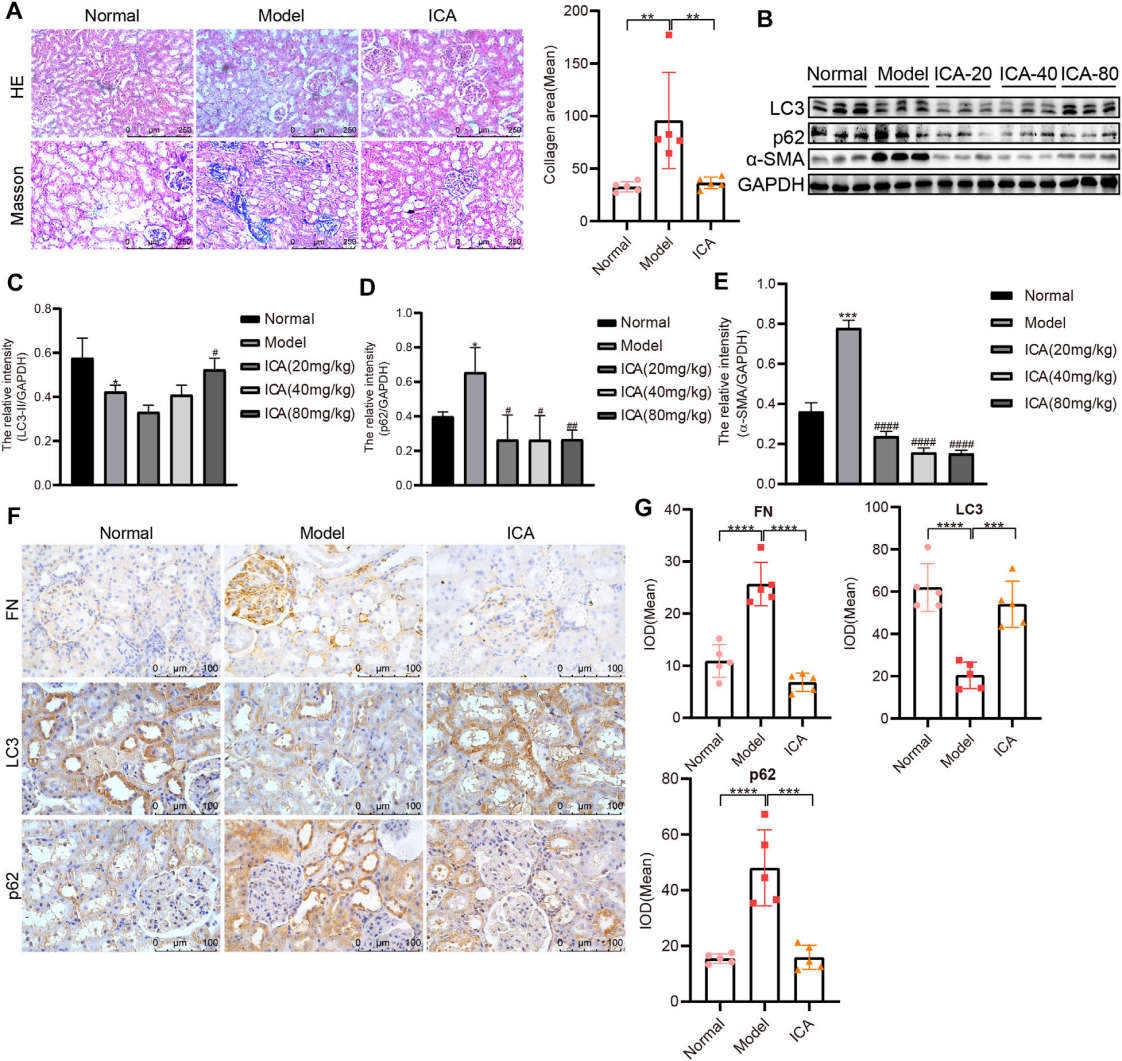
ICA promotes autophagy and alleviates renal fibrosis in T2DN rats **(A)** Representative image of HE and Masson staining in Normal, Model and ICA + Model group (Scale bar: 250 μm; original magnification: ×200). ICA was administered at 80 mg/kg. Error bars represent SD; *n* = 5; **p < 0.01 **(B)** Results of LC3, p62, and *α*-SMA, as determined by Western blot **(C–E)** Representative Western blot analysis of LC3, p62, and *α*-SMA expression in rat renal tissues. Error bars represent SD; *n* = 3; *p < 0.05, ***p < 0.001, *vs* Normal; ^#^ *p* < 0.05, ^##^ *p* < 0.01, ^####^ *p* < 0.0001 *vs* Model **(F)** Immunohistochemical staining of FN, LC3, and p62 in rat renal tissues (Scale bar: 100 μm; original magnification: ×400) **(G)** The mean IOD of LC3, p62, and FN expression was analyzed by IPP software. Error bars represent SD; *n* = 5; ***p < 0.001, ****p < 0.0001.

HE staining of renal tissues exposed increased renal injury in the model group; after ICA treatment, the renal injury was improved. The Masson staining in the model group showed increased collagen deposition, and ICA decreased collagen deposition (Figure 2A). Accumulation of *a*-SMA and p62 was increased compared to that in normal rats; however, expression of *α*-SMA and p62 was extremely reduced in DN + ICA. Moreover, the accumulation points of LC3 were significantly reduced in the renal tissues of DN rats, and ICA rescued LC3 aggregation (Figures 2B–E). The results of immunohistochemical staining (IHC) also showed that ICA significantly dampened the increase in FN and p62 expression, and the decrease in LC3 expression in the diabetic rat kidney (Figures 2F, G). The specificity of anti-FN, p62, and LC3 antibodies was verified by Western blot (Supplementary Figure 5A–C). Overall, these results suggest that ICA reverses the effects of autophagy and fibrosis in T2DN rats.

### ICA Alleviates HG-Induced Fibrosis by Inducing Autophagy in HK-2 and NRK-49F Cells

**FIGURE 3:**
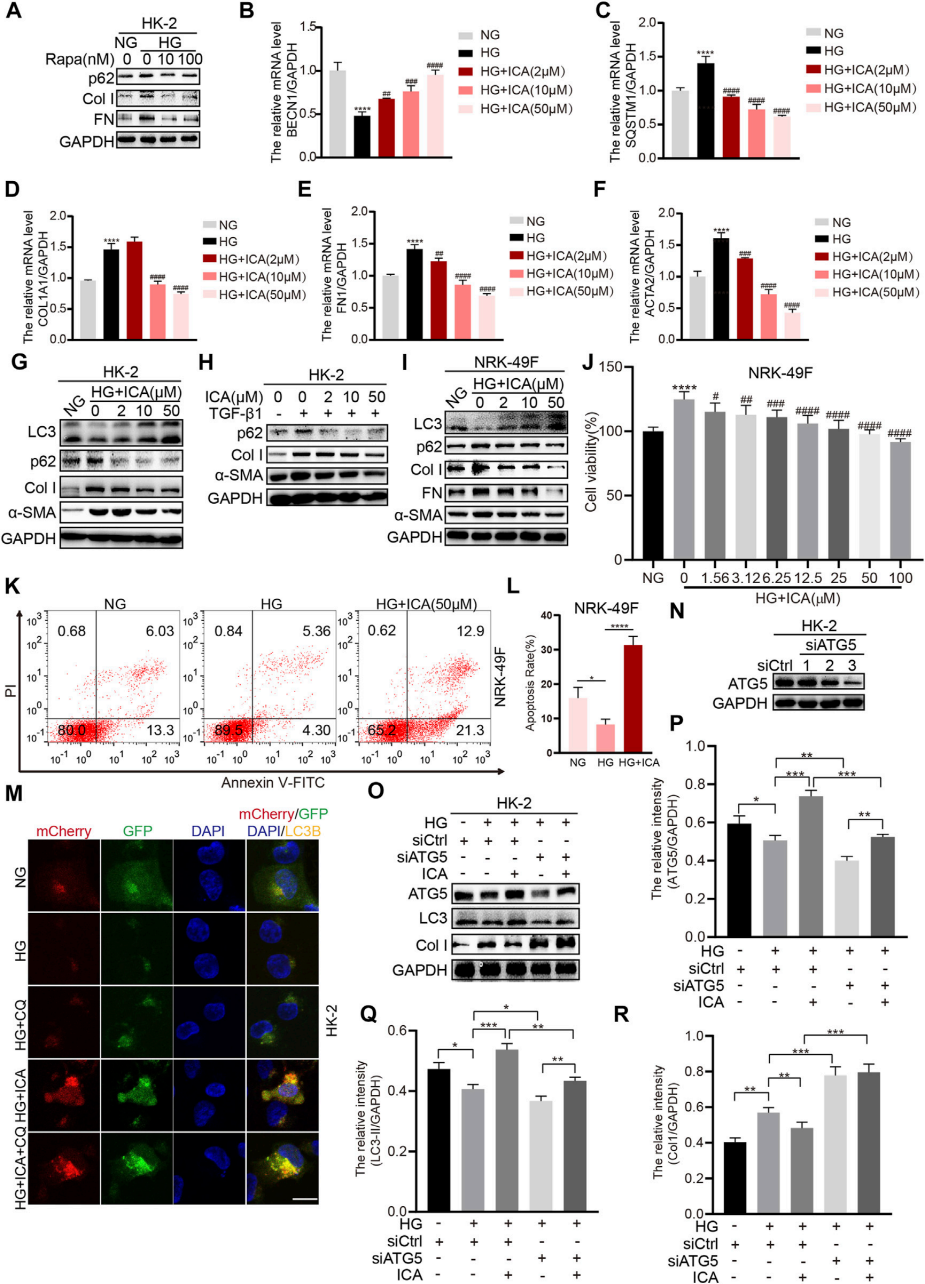
ICA alleviates fibrosis by inducing autophagy in HK-2 and NKR-49F cells **(A)** HK-2 cells were treated with HG and rapamycin for 48 h, then the expression of p62, Col I, and FN was determined by Western blot **(B–F)** The relative levels of *BECN1*, *SQSTM1*, *COL1A1*, *ACTA2*, and *FN1* were detected by real-time PCR after HK-2 cells were stimulated with HG and various concentrations of ICA for 48 h ****p < 0.0001 *vs* NG; ^##^ *p* < 0.01, ^###^ *p* < 0.001, ^####^ *p* < 0.0001 *vs* HG **(G–I)** Protein expression of autophagy and fibrosis in HK-2 and NRK-49F cells after incubation with HG and ICA (2, 10, 50 μM) or TGF-β1 (5 ng/ml) for 48 h **(J)** The cell viability was analyzed in NRK-49F cells after incubation with HG and various concentrations of ICA for 48 h *n* = 6; ****p < 0.0001 vs NG, ^#^ *p* < 0.05, ^##^ *p* < 0.01, ^###^ *p* < 0.001, ^####^ *p* < 0.0001 *vs* HG **(K–L)** The total apoptosis rates detection and analysis by flow cytometry after NRK-49F cells were treated with HG and ICA for 48 h (mean ± SD; *n* = 3; *p < 0.05, ****p < 0.0001) (M) HK-2 cells were infected with an LC3B-encoding adenovirus, treated with HG (30 mM), ICA (50 μM) or CQ (5 μM). Scale bar: 50 μM, original magnification: ×630. Representative immunofluorescence images are shown (N–R) HK-2 cells were transfected with siATG5 or NC, then treated with or without ICA (50 μM). The expression of ATG5, LC3, and Col I was evaluated and analyzed by the ChemiScope analysis software (*p < 0.05, **p < 0.01, ***p < 0.001, ****p < 0.0001).

A previous study indicated that hyperglycemia can induce extracellular matrix accumulation of renal tubular epithelial cells and renal interstitial fibroblasts, which is a vital step in tubulointerstitial fibrosis (He et al., 2016; Jao et al., 2019). The HK-2 cells were co-treated with high glucose (HG) and rapamycin, and the results showed that rapamycin could weaken HG-induced fibrosis by increasing autophagy (Figure 3A). Treatment with HG significantly increased the mRNA levels of *SQSTM1*, *COL1A1*, *FN1,* and *ACTA2*, and decreased the mRNA levels of *BECN1*, whereas ICA decreased *SQSTM1*, *COL1A1*, *FN1,* and *ACTA2* expression and increased *BECN1* expression (Figures 3B–F). In addition, treatment with ICA also decreased HG- or TGF-β1-induced p62, *α*-SMA, FN and Collagen I (Col I) expression and increased LC3 expression at the protein level (Figures 3G–I). ICA also inhibited cell viability and increased the cell apoptosis rate in NRK-49F cells under HG conditions (Figures 3J–L), indicating that ICA inhibited HG-induced activation of renal interstitial fibroblasts. To provide evidence that ICA induces autophagic flux, we infected HK-2 cells with an adenovirus encoding mCherry-GFP-LC3B which results in both green and red fluorescence. Analysis of the distribution of the mCherry-GFP-LC3B fusion protein in ICA-treated HK-2 cells exhibited increased red and yellow fluorescence, and compared with HG + ICA, the red and yellow fluorescence in HG + ICA + CQ was also increased, indicating that autophagic flux had increassed (Figure 3M). Then, we applied Atg5-siRNA to explore the relationship between autophagy and fibrosis under HG and HG + ICA condition. After Atg5-siRNA transfection (Figure 3N), autophagy was inhibited. Although the improved effect of ICA on fibrosis still existed, it was impaired (Figure 3O–R). All of these results indicate that ICA relieves fibrosis by enhancing autophagy.

### ICA Induces Autophagy and Reduces Fibrosis Dependently on Androgen Receptor

**FIGURE 4:**
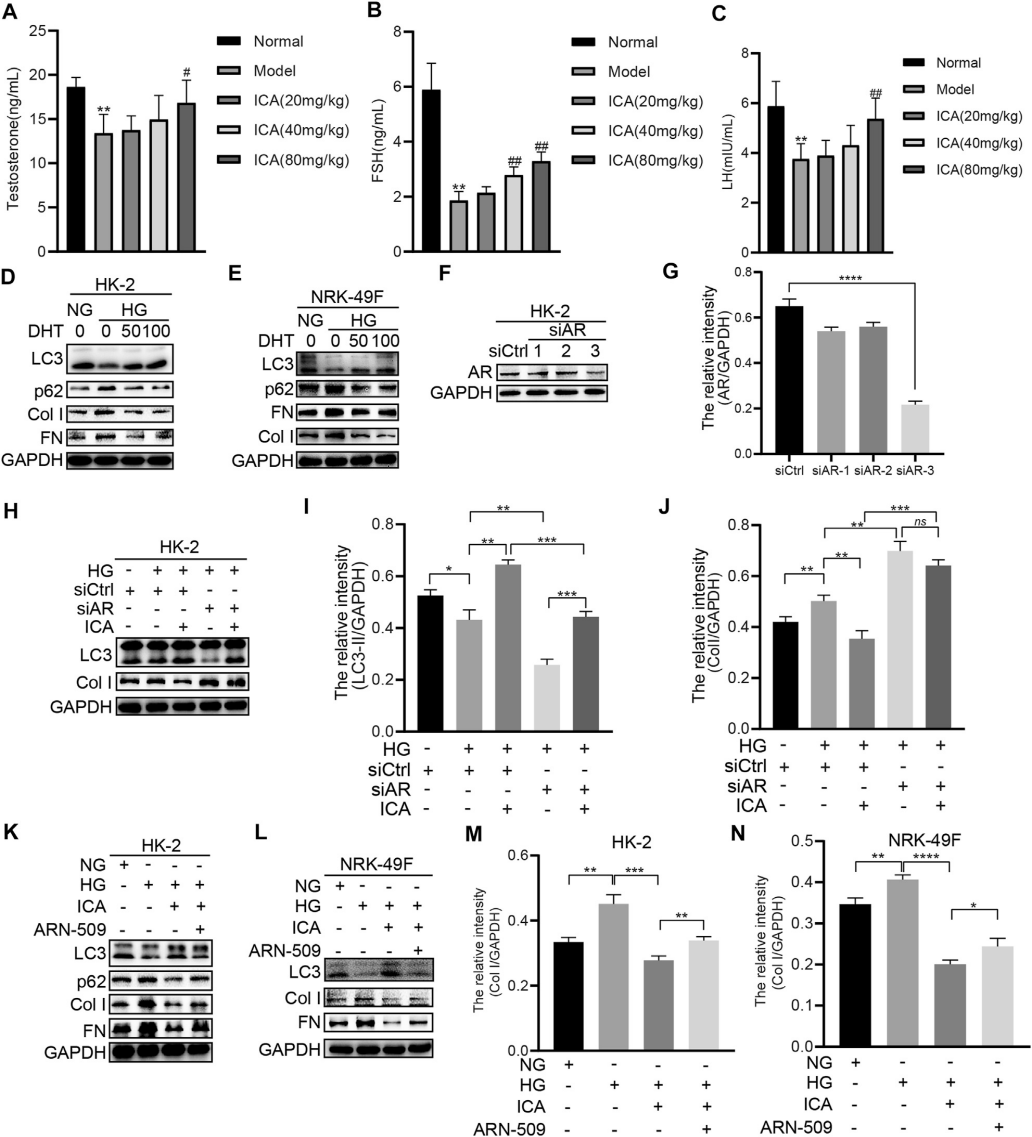
ICA induces autophagy and reduces fibrosis by regulating AR **(A–C)** The serum testosterone, FSH, and LH concentrations were detected by ELISA. Data are expressed as mean ± SD (*n* = 8). **p < 0.01, *vs* the Normal group; ^#^ *p* < 0.05, ^##^ *p* < 0.01, vs the Model group **(D–E)** HK-2 and NRK-49F cells were treated with HG and DHT for 48 h, then the expression of LC3, p62, Col I, and FN was detected by Western blot **(F–J)** HK-2 cells were transfected with siAR or NC, then treated with or without ICA (50 μM), the expression of AR, LC3, and Col I was evaluated by Western blot and analyzed by the ChemiScope analysis software (*p < 0.05, **p < 0.01, ***p < 0.001, ****p < 0.0001) (K–N) HK-2 and NRK-49F cells were treated with HG, ICA (50 μM) or ARN-509 (80 nM), and the expression of LC3, p62, Col I, and FN was detected by Western blot and the relative intensity of Col I was analyzed by the ChemiScope analysis software (*p < 0.05, **p < 0.01, ***p < 0.001, ****p < 0.0001).

The levels of testosterone, FSH, and LH were all remarkably decreased in the DN model group compared to the normal group, and ICA dose dependently increased those values (Figures 4A–C). DHT (Dihydrotestosterone) impaired HG-induced p62, Collagen I, and FN expression, and restored HG-reduced LC3 expression (Figures 4D, E). Androgen exerts its function *via* the androgen receptor (AR), which is a transcription factor that belongs to the nuclear receptor superfamily. If AR is absent or inhibited, androgens cannot stimulate cells or tissues (Kono et al., 2017). Therefore, we next determined whether ICA-regulated autophagy and fibrosis was dependent on AR. ICA, siRNA for AR, and a selective and competitive antagonist of AR (ARN-509) were added to HK-2 or NRK-49F cells under HG condition. The HK-2 cells were cultured with endogenous AR inhibition by transfecting a small interference RNA (siRNA) targeting the common region of AR (Figures 4F, G). In cells transfected with valid AR siRNA and kept in HG condition, autophagy was inhibited and the fibrosis was increased, and the effect of ICA on autophagy and fibrosis was weakened (Figures 4H–J). Moreover, compared to ICA, the expression of LC3, p62, Col I, and FN in ICA + ARN-509 was significantly increased (Figure 4K–N). These data suggest that the autophagy induction and renal fibrosis improvement by ICA treatment was dependent on AR.

### DHT Induces Autophagy and Reduces Fibrosis by Decreasing miR-192-5p

**FIGURE 5:**
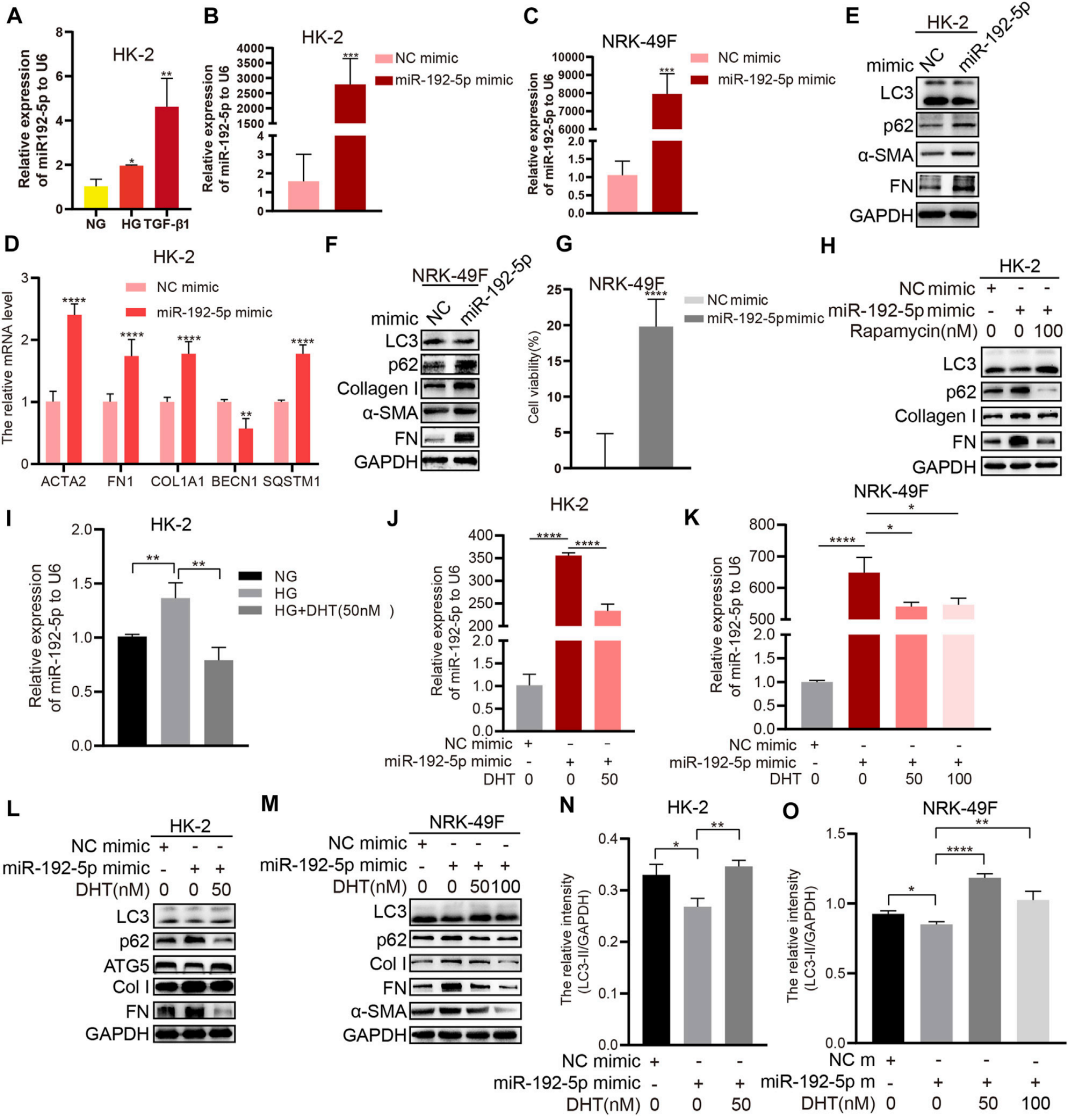
DHT induces autophagy and reduces fibrosis by down-regulating miR-192-5p expression **(A)** Real-time PCR detection of miR-192-5p after HK-2 cells stimulated with HG and TGF-β1. *n* = 3; *p < 0.05, **p < 0.01 **(B–C)** Relative expression of miR-192-5p in HK-2 and NRK-49F cells transfected with miR-192-5p mimic. ***p < 0.001 **(D)** Relative mRNA levels of *BECN1*, *SQSTM1*, *COL1A1*, *ACTA2*, and *FN1* in HK-2 cells transfected with miR-192-5p mimic. **p < 0.01, ****p < 0.0001 **(E–F)** The expression of LC3, p62, *α*-SMA, Col I, and FN in HK-2 and NRK-49F cells transfected with miR-192-5p mimic **(G)** The cell viability in NRK-49F cells transfected with miR-192-5p mimic. ****p < 0.0001 **(H)** HK-2 cells were transfected with miR-192-5p mimic and treated with rapamycin, the expression of LC3, p62, Col I, and FN was detected **(I)** Relative expression of miR-192-5p in HK-2 cells treated with HG and DHT. **p < 0.01 **(J–K)** Relative expression of miR-192-5p in HK-2 and NRK-49F cells transfected with miR-192-5p mimic and treated with DHT. *p < 0.05, ****p < 0.0001 **(L–O)** The expression of LC3, p62, ATG5, *α*-SMA, Col I, and FN in HK-2 and NRK-49F cells transfected with miR-192-5p mimic and treated with DHT was determined by Western blot and the relative intensity of LC3-II was analyzed by the ChemiScope analysis software (*p < 0.05, **p < 0.01, ****p < 0.0001).

HG and TGF-β1 upregulated miR-192-5p expression in HK-2 cells (Figure 5A). We transfected HK-2 cells or NRK-49F cells with a miR-192-5p mimic or negative control (NC) mimic and found the overexpression of miR-192-5p (Figures 5B, C) increased the mRNA levels of *ACTA2*, *FN1*, *COL1A1,* and *SQSTM1* and decreased *BECN1* mRNA expression in HK-2 cells (Figure 5D). Consistently, the forced miR-192-5p expression also enhanced the protein levels of *α*-SMA, FN, Col I, and p62, and downregulated LC3 expression both in HK-2 and NRK-49F cells (Figures 5E, F), indicating the effect of miR-192-5p on inhibiting autophagy and promoting fibrosis. Moreover, forced miR-192-5p expression in NRK-49F cells promoted the cell viability (Figure 5G), indicating that miR-192-5p promotes the activation of NRK-49F cells. Additionally, rapamycin reversed miR-192-5p overexpression-mediated fibrosis and autophagy (Figure 5H), suggesting that inducing autophagy could alleviate miR-192-5p-induced fibrosis. Compared to HG alone, miR-192-5p expression in HG + DHT was significantly reduced (Figure 5I). Notably, miR-192-5p levels were increased after transfecting with miR-192-5p mimic, but were reduced remarkably after DHT treatment (Figures 5J, K). Moreover, levels of p62, Col I, FN, and *α*-SMA were significantly decreased, and LC3 and ATG5 was increased in miR-192-5p mimic + DHT group compared to those in miR-192-5p mimic group (Figure 5L–O). Therefore, we consider that DHT regulates the autophagy and fibrosis through decreasing miR-192-5p in HK-2 and NRK-49F cells.

### ICA Induces Autophagy and Reduces Fibrosis by Decreasing miR-192-5p

**FIGURE 6:**
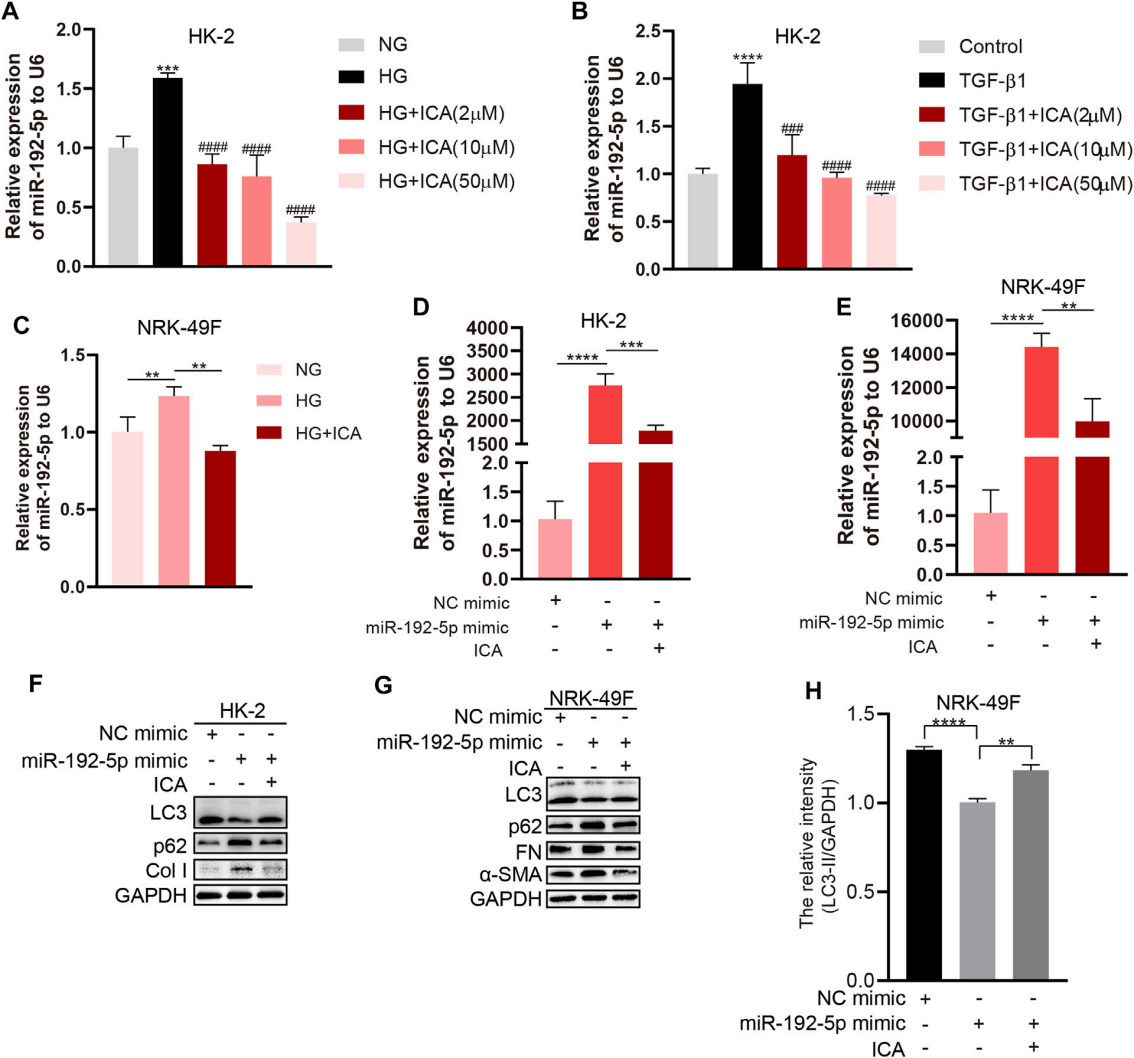
ICA induces autophagy and reduces fibrosis by down-regulating miR-192-5p expression **(A–B)** Relative expression of miR-192-5p in HK-2 cells treated with HG, TGF-β1 and ICA. ***p < 0.001, ****p < 0.0001 *vs* NG, ^###^ *p* < 0.001, ^####^ *p* < 0.0001 *vs* HG **(C)** Relative expression of miR-192-5p in NRK-49F cells treated with HG (30 mM) and ICA (2 μM). **p < 0.01 **(D–E)** Relative expression of miR-192-5p in HK-2 and NRK-49F cells transfected with miR-192-5p mimic and treated with ICA (2 μM). **p < 0.01, ***p < 0.001, ****p < 0.0001 **(F–G)** The expression of LC3, p62, *α*-SMA, Col I and FN in HK-2 and NRK-49F cells transfected with miR-192-5p mimic and treated with ICA (2 μM) **(H)** The relative intensity of LC3-II in NRK-49F cells was analyzed by the ChemiScope analysis software, **p < 0.01, ****p < 0.0001.

The miR-192-5p level was significantly increased with HG or TGF-β1 treatment alone but reduced in HG + ICA or TGF-β1 + ICA in HK-2 cells (Figures 6A–C). In addition, miR-192-5p was significantly increased after transfecting with miR-192-5p mimic but reduced after ICA treatment (Figures 6D, E). We found that levels of p62, Col I, FN, and *α*-SMA were significantly decreased, and LC3 was increased in miR-192-5p mimic + ICA compared to those in miR-192-5p mimic (Figures 6F–H). All of these results indicate that ICA regulates autophagy and fibrosis by decreasing miR-192-5p.

### ICA and DHT induces autophagy and reduces fibrosis through the miR-192-5p/GLP-1R pathway

**FIGURE 7:**
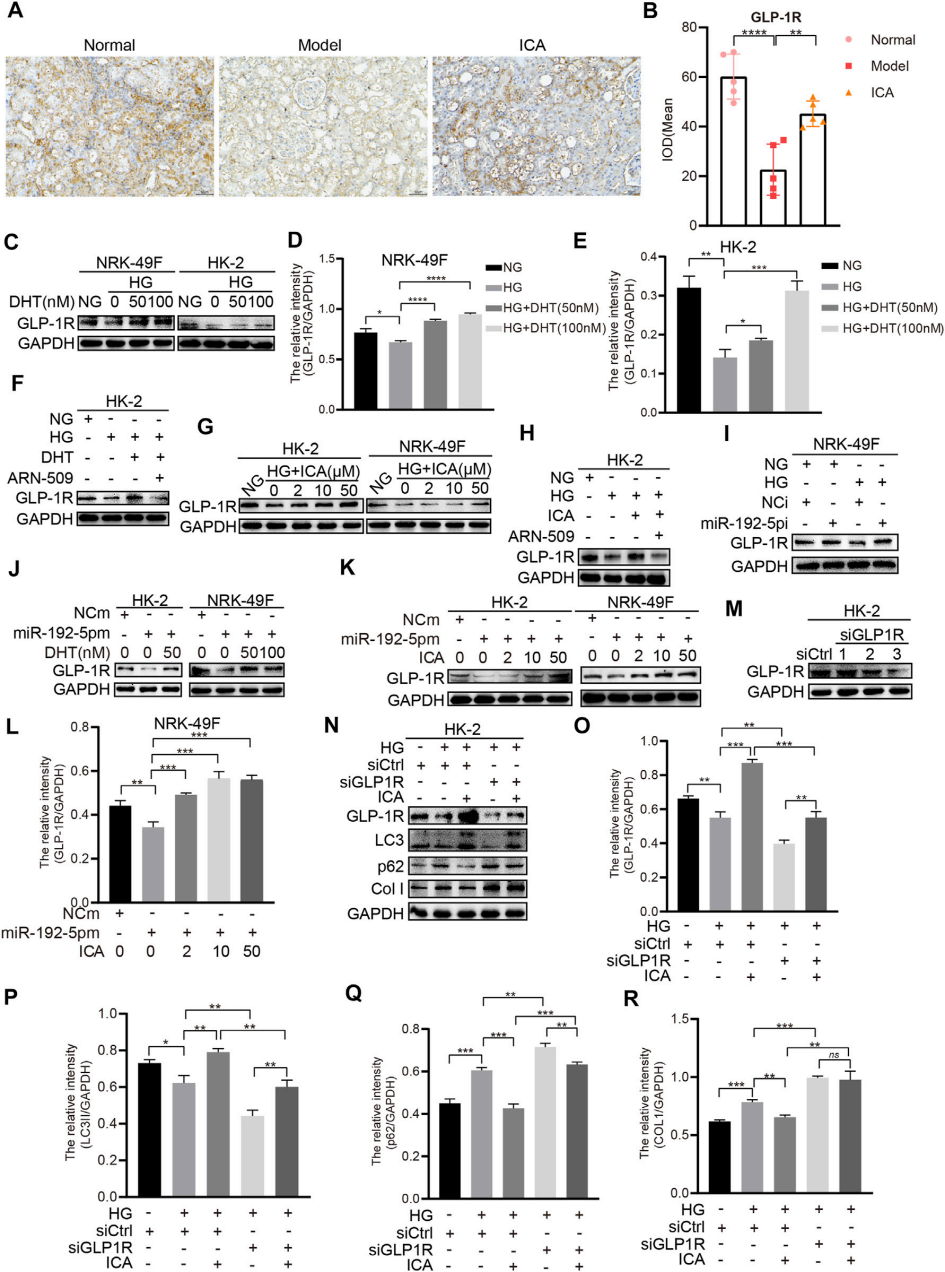
ICA and DHT induce autophagy and reduce fibrosis through miR-192-5p/GLP-1R pathway **(A)** Immunohistochemical staining of GLP-1R in rats renal tissues (Scale bar: 50 μm; original magnification: ×200) **(B)** The mean IOD of GLP-1R expression was analyzed by IPP software. Error bars represent SD; *n* = 5; **p < 0.01, ****p < 0.0001 **(C–E)** HK-2 and NRK-49F cells were treated with HG and DHT for 48 h, then the expression of GLP-1R was detected by Western blot and analyzed by the ChemiScope analysis software (*p < 0.05, **p < 0.01, ***p < 0.001, ****p < 0.0001) **(F)** HK-2 cells were treated with HG, DHT or ARN-509, and the expression of GLP-1R was detected **(G)** The expression of GLP-1R in HK-2 and NRK-49F cells treated with HG and ICA **(H)** HK-2 cells were treated with HG (30 mM), ICA (50 μM) or ARN-509, and the expression of GLP-1R was detected by Western blot **(I)** GLP-1R expression in NRK-49F cells transfected with miR-192-5p inhibitor and exposed to HG was analyzed by Western blot **(J)** GLP-1R expression in HK-2 and NRK-49F cells transfected with miR-192-5p mimic and treated with DHT **(K)** GLP-1R expression in HK-2 and NRK-49F cells transfected with miR-192-5p mimic and treated with ICA **(L)** The relative intensity of GLP-1R was analyzed by the ChemiScope analysis software (**p < 0.01, ***p < 0.001) **(M–R)** The expression of GLP-1R, LC3, p62, and Col I in HK-2 cells transfected with siGLP-1R and treated with HG and ICA (50 μM) was evaluated by Western blot and analyzed by the ChemiScope analysis software (*p < 0.05, **p < 0.01, ***p < 0.001).

**FIGURE 8:**
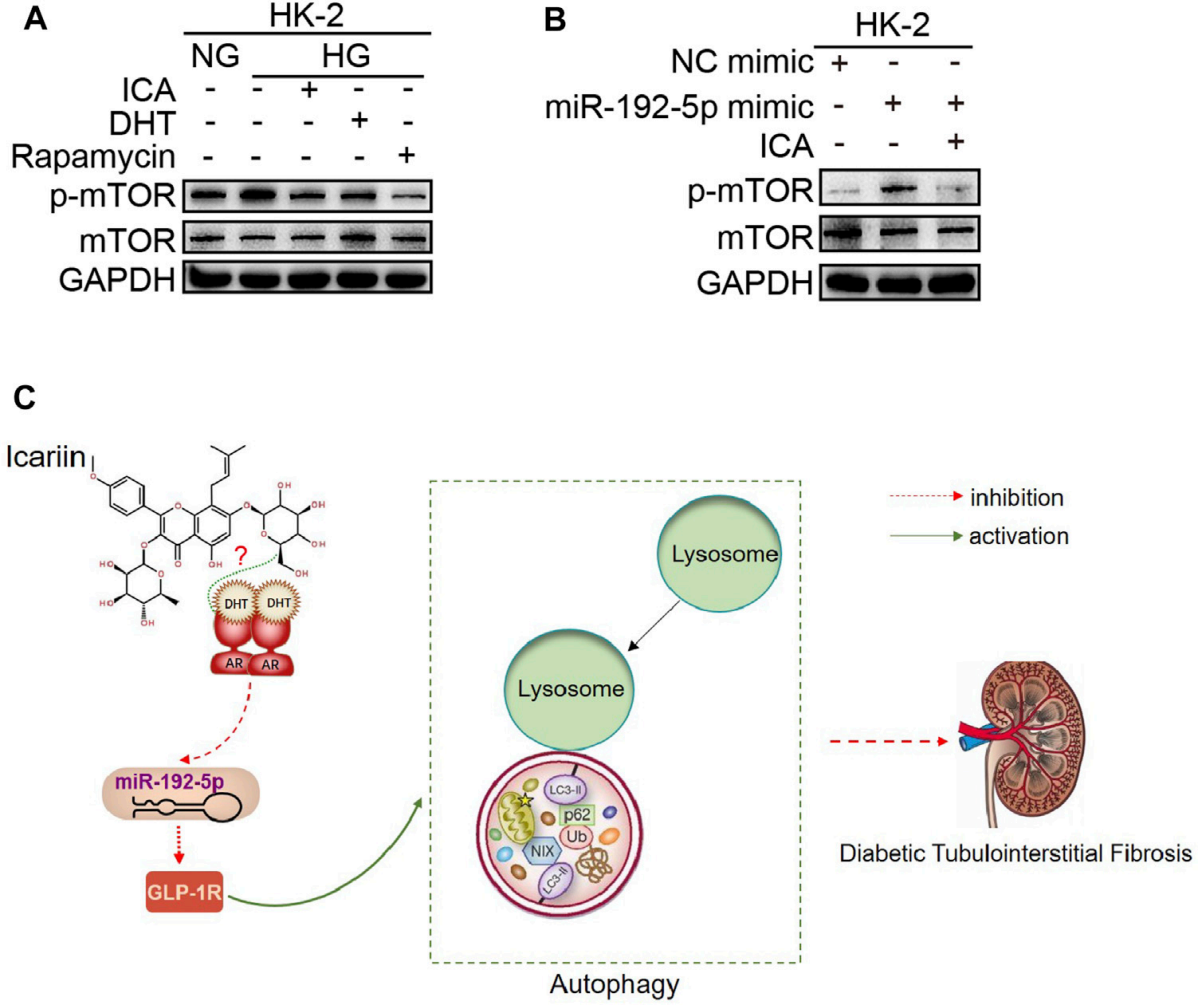
ICA and DHT induce autophagy by reducing phosphorylated mTOR (*p*-mTOR) expression **(A)** The HK-2 cells were stimulated with HG and treated respectively with ICA (50 μM), DHT (100 nM) and rapamycin (100 nM) for 48 h, the expression of *p*-mTOR and mTOR was detected **(B)** The *p*-mTOR and mTOR expression in HK-2 cells transfected with miR-192-5p mimic and treated with ICA (2 μM) **(C)** Mechanisms for the anti-diabetic tubulointerstitial fibrosis effect of ICA.

We next determined the effect of DHT and ICA on the miR-192-5p/GLP-1R pathway in renal tissues from rats and in HK-2 cells. The GLP-1R expression in T2DN rats was obviously downregulated compared with that in normal rats, and ICA restored the GLP-1R expression (Figures 7A, B). The specificity of anti-GLP-1R antibody was verified by Western blot (Supplementary Figure 5D). Interestingly, the expression of GLP-1R was decreased after HG stimulation, and could be reversed by DHT (Figures 7C–E). Furthermore, compared to HG + DHT, the expression of GLP-1R in HK-2 cells treated with HG + DHT + ARN-509 returned to the level in HG treatment group (Figure 7F). Moreover, compared to HG, ICA increased GLP-1R expression under the HG condition (Figure 7G). Compared to HG + ICA, the expression of GLP-1R in HG + ICA + ARN-509 was obviously downregulated (Figure 7H). Additionally, the miR-192 inhibitor reversed HG induced GLP-1R expression (Figure 7I), and forced miR-192-5p expression induced reduced GLP-1R levels could be reversed by DHT (Figure 7J). Noteworthy, the expression of GLP-1R was significantly upregulated in miR-192-5p mimic + ICA group compared with that in miR-192-5p mimic group (Figures 7K–L), suggesting that ICA increased GLP-1R expression by decreasing miR-192-5p expression. To down-regulate GLP-1R expression, we tested a panel of siRNAs for down-regulation of GLP-1R expression (Figure 7M). A valid siRNA was selected and used to suppress GLP-1R expression in subsequent experiments. In cells with down-regulated GLP-1R under HG conditions, the protein levels of p62 and Col I were significantly increased, and LC3 expression was decreased compared with that in negative control group. In addition, the effect of ICA on expression of these proteins was weakened (Figures 7N–R). Moreover, ICA and DHT significantly reduced HG-induced phosphorylated mTOR (*p*-mTOR) expression (Figure 8A), and ICA also inhibited miR-192-5p overexpression induced *p*-mTOR expression (Figure 8B). Collectively, we consider that ICA and DHT induces autophagy and reduces fibrosis by regulating miR-192-5p/GLP-1R pathway.

## Discussion

Fibrosis is crucial in the process of DN eventually developing into renal failure (Zeng et al., 2019). Therefore, the prevention and treatment of renal fibrosis is very important for patients with DN (Djudjaj and Boor, 2019). In this study, for the first time, we found that ICA and DHT downregulated miR-192-5p and upregulated GLP-1R expression, which is a novel mechanism by which ICA induces autophagy and alleviates tubulointerstitial fibrosis.

ICA is the main active ingredient of total flavonoids in the epimedium plant of Berberiaceae, which has multiple pharmacological properties, such as promoting bone metabolism, anti-cancer effects, and regulating immune, endocrine, and reproductive systems (He et al., 2020a). Previously, studies have reported that ICA protects against renal injury and fibrosis in an experimental unilateral ureteral obstruction (UUO) model Chen et al. (2019) and ameliorates diabetic cardiomyopathy in db/db mice (Ni et al., 2020). Our and other research groups have confirmed that ICA exerted preventive function of kidney disease in STZ-induced type 1 diabetic rats (Qi et al., 2011; Wang et al., 2020). However, the effect of ICA on T2DN is yet unclear. In this study, we explored the effect of ICA on T2DN rats using a high-sugar and high-fat diet and peritoneal injection of STZ, which is a recognized model of T2DN (Lin et al., 2010; Wen et al., 2012; Li et al., 2019). The results in this study verified that ICA improved renal function by decreasing the levels of BUN, serum creatinine, and urinary protein and increasing creatinine clearance in rat T2DN model. The mean levels of UCr in the high dose of ICA group were increased over the model group; however, due to the higher SD value, the difference between the two groups was not significant. Although serum creatinine and urine creatinine are all indicators representing renal function, because urine creatinine fluctuates greatly, the level of blood creatinine is mainly used in the clinic to determine renal function. Our results showed that ICA obviously decreased the level of serum creatinine and increased the creatinine clearance, indicating the improvement of ICA on the renal function. In addition, ICA attenuated renal fibrosis by decreasing the expression of *a*-SMA and FN, reduced renal tissues inflammation, collagen deposition and fibrogenesis in rat T2DN model. These results suggest that ICA has the effect of ameliorating renal function of T2DN by inhibiting fibrosis.

Tubulointerstitial fibrosis represents a common final pathway for all kidney diseases, including DN, that lead to progressive renal injury and is a better predictor of renal disease progression than glomerular injury (Zeisberg and Neilson, 2010; He et al., 2020b). However, the therapies treating tubulointerstitial fibrosis are insufficient. In this study, we found that ICA inhibited activation of NRK-49F cells and reduced HG-induced expression of collagen I, *α*-SMA, and FN in HK-2 and NRK-49F cells that involves tubulointerstitial fibrosis. Therefore, ICA plays an effective role against renal fibrosis in the treatment of diabetic kidney disease, and the effect may be associated with the alleviation of tubulointerstitial fibrosis. How ICA ameliorates tubulointerstitial fibrosis in diabetic kidney disease is yet unclear.

Autophagy is a highly conserved, catabolic process in which superfluous or damaged organelles and protein aggregates are degraded in lysosomes (Galluzzi et al., 2017). Notably, autophagy was inhibited in DN models and in human diabetic kidneys (Kume et al., 2012; Yang et al., 2018). Moreover, recent studies have shown that activation of autophagy can inhibit fibrosis and prevent the progression of DN (Huang et al., 2019). Generally, LC3-II formation is recognized as a marker of autophagosomes in cell or animal experiments (Mizushima et al., 2010). In this study, we demonstrated that ICA induced autophagy by increasing the expression of LC3-II and decreasing the p62 expression, both in the renal tissues of STZ-induced type 2 diabetes rat models and in HG-treated HK-2 and NRK-49F cells. Moreover, by transfecting with siATG5, we determined ICA reduced fibrosis by inducing autophagy. Therefore, we consider that ICA may attenuate tubulointerstitial fibrosis by activating autophagy. It has been reported that ICA promoted androgen production and upregulated the expression of transcription factor SF-1 (adrenal 4-binding protein) and steroid-producing enzymes Chen et al. (2014), Sun et al. (2019), and ICA improves the physiological state of the testicles and increases the circulating levels of testosterone (Zhang and Yang, 2006). Thence, ICA has androgen-like effects and can ameliorate sexual dysfunction (Koizumi et al., 2010; Liu et al., 2021). Interestingly, studies have suggest a gender difference in type 2 diabetes, with a higher prevalence and increased type 2 diabetes risk in males (Flegal et al., 2010; Flegal et al., 2010; Kautzky-Willer et al., 2016; Aregbesola et al., 2017). Though most studies examining sex hormones have focused on type 2 diabetes, there is no clear link between testosterone levels and the development of type 1 diabetes (Biswas et al., 2012; Kelsey et al., 2016). Notably, patients with T2DN are predominantly male, and compared with female T2DN patients, the incidence of male T2DN patients has increased Flegal et al. (2010), Shepard (2019), and are more likely to have persistent microalbuminuria and large proteinuria (Aregbesola et al., 2017). For this reason, we chose the male T2DN rats as a research model to explore the effect of ICA on T2DN and sex hormone levels. Moreover, studies in humans have shown that diabetes is associated with an imbalance in sex hormone levels. Namely, males have low testosterone and high estradiol levels Grossmann et al. (2009), whereas females exhibit low estradiol and higher testosterone levels (Salonia et al., 2006). These studies suggest that sex hormones play different roles in T2DN of males and females. In this research, we found that the levels of testosterone, LH, and FSH were decreased significantly in the male T2DN model group compared to the male normal group; however, ICA could obviously restore these values compared with that in T2DN model group. We also investigated the effect of DHT, the main androgen in humans, on autophagy and fibrosis under HG condition, and found that DHT reversed the effect of HG on autophagy and fibrosis. By using ARN-509 and siRNA for AR, we determined that the effect of ICA induced autophagy and reduced diabetic renal tubulointerstitial fibrosis was AR-dependent. Previous research has demonstrated that ICA stimulates release of NO by AR-dependent activation of eNOS in human umbilical vein endothelial cells Biswas et al. (2012), Kelsey et al. (2016), indicating the involvement of AR in ICA regulated pharmacology. Therefore, ICA alleviates T2DN and diabetic renal tubulointerstitial fibrosis by exerting an androgen-like effect in male DN rats. On the other hand, icariin was previously shown to exert estrogen-like protective effects on bone in ovariectomized (OVX) mice, suggesting that its estrogenic effects in rat osteoblastic cells were ER-dependent (Zhou et al., 2021). Icariin exerts estrogen-like activity in ameliorating experimental autoimmune encephalomyelitis via mediating estrogen receptor β in female mice (Wei et al., 2016). These studies suggest that ICA has estrogen-like activity, and we speculate that ICA may alleviate T2DN and diabetic renal tubulointerstitial fibrosis by exerting estrogen-like activity in females. The effect and mechanism of ICA alleviating T2DN in females warrants further research.

Next, we investigated how ICA and DHT regulates autophagy and fibrosis. GLP-1R is highly expressed in the kidney, especially in renal tubular epithelial cells (Zhao et al., 2015). Research has demonstrated that *Glp1r* mRNA levels positively correlates with the serum testosterone concentrations in normal male mice and in diabetic models (Zhu et al., 2019). MicroRNAs (miRNAs) are a class of small non-coding RNAs that regulate gene expression by either downregulating mRNA levels or directly repressing translation of genes, and miRNAs are attractive therapeutic targets for the treatment of diabetes mellitus and its complications (Regazzi, 2018). Of note, research has demonstrated that inhibiting miR-192 can ameliorate diabetes and diabetic nephropathy (Putta et al., 2012; Ebadi et al., 2019). Moreover, miR-192 targets GLP-1R by binding to its 3′-UTR and promotes renal fibrosis through reducing GLP-1R expression (Jia et al., 2018). A search of the TargetScanHuman online database showed that GLP-1R can only be regulated by miR-192-5p/215-5p. Therefore, we considered that ICA and DHT may regulate autophagy and fibrosis *via* miR-192/GLP-1R pathway. In this study, we found that DHT and ICA could reverse HG and miR-192-5p mimic induced upregulation of miR-192-5p, and dampen the effect of HG and miR-192-5p overexpression-regulated autophagy and fibrosis. We also found that under HG conditions or when miR-192-5p is overexpressed, DHT and ICA increase GLP-1R expression in both HK-2 and NRK-49F cells. Moreover, ICA upregulated GLP-1R expression by regulating AR; after silencing GLP-1R, the effect of ICA on HG-regulated autophagy and fibrosis was weakened. In this research, we found that GLP-1R expression was significantly decreased in T2DN model group compared to the normal group, and ICA restored the GLP-1R expression relative to the model group. It has been reported that GLP-1 alleviates diabetic kidney disease by significantly decreasing urinary albumin and ameliorating renal pathological changes *in vivo*, and improves autophagy through mTOR signaling pathway (Yang et al., 2020). We demonstrated that ICA inhibited HG and miR-192-5p overexpression induced *p*-mTOR expression, further indicating the effect of ICA activating autophagy.

In conclusion, these findings indicate that ICA and DHT alleviates diabetic renal tubulointerstitial fibrosis by restoring autophagy through the miR-192-5p/GLP-1R pathway and provide novel options for the treatment of diabetic renal fibrosis (Figure 8C).

## Data Availability

The raw data supporting the conclusions of this article will be made available by the authors, without undue reservation.
