# Supplementary material for: Icariin Ameliorates Diabetic Renal Tubulointerstitial Fibrosis by Restoring Autophagy via Regulation of the miR-192-5p/GLP-1R Pathway
Source: Front Pharmacol. 2021 Jul 19;12:720387. doi: 10.3389/fphar.2021.720387 (PMC8326523; doi:10.3389/fphar.2021.720387)
Supplement: Supplementary file 1 [file DataSheet1.docx]

**Supplementary Materials**


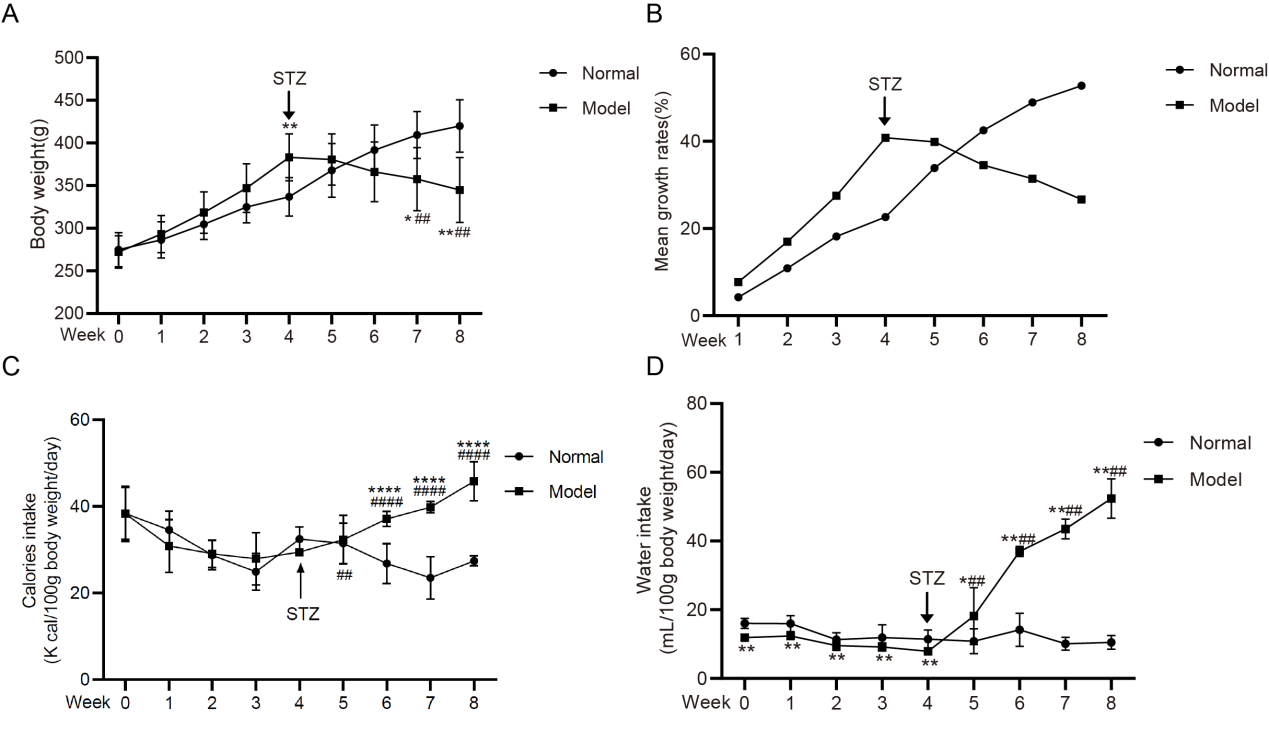


Figure S1 (A) Body weight, (B) the mean growth rates ((week n- week 0)/week 0*100%), (C) Diet changes, (D) Changes of water intake before ICA administration. Data are expressed as mean ± SD. **P*<0.05, ***P*<0.01, *****P*<0.0001 *vs* Normal group, ^##^*P*<0.01, ^####^*P*<0.0001 *vs* Week 4 (Normal, *n*=8; Model, *n*=40-42).


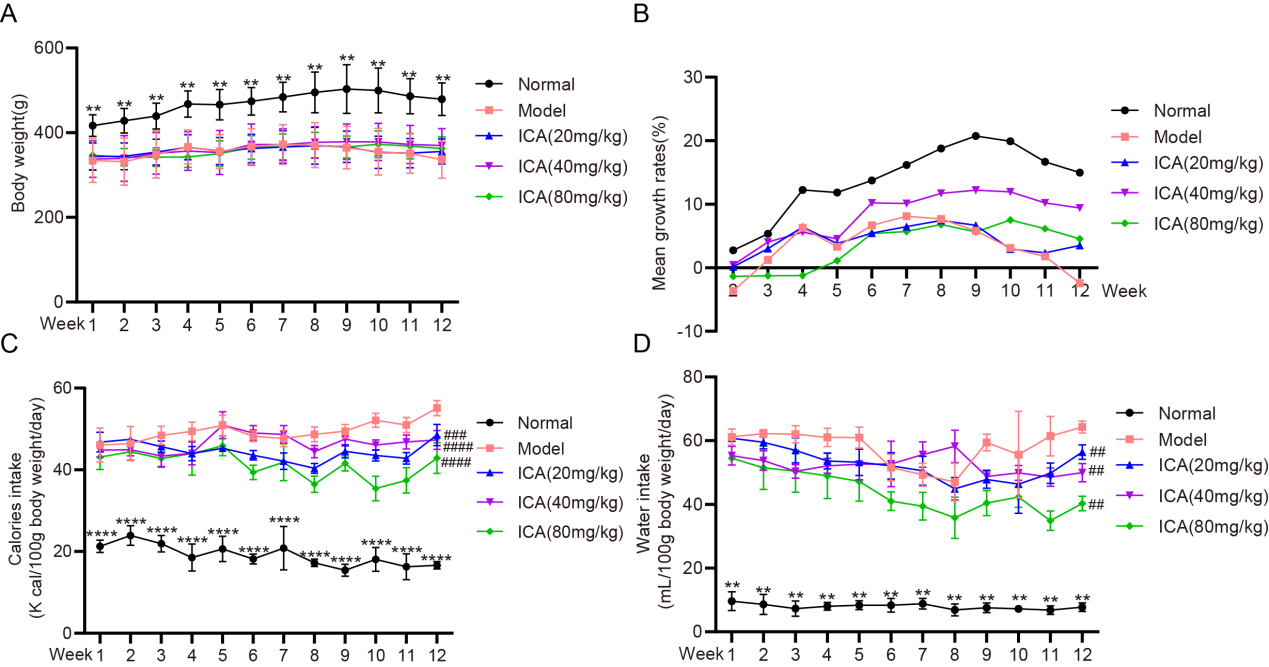


Figure S2 (A) Body weight, (B) the mean growth rates ((week n- week 1)/week 1*100%), (C) Diet changes, (D) Changes of water intake after ICA administration. Data are expressed as mean ± SD. ***P*<0.01, *****P*<0.0001 *vs* Normal group, ^##^*P*<0.01, ^###^*P*<0.001, ^####^*P*<0.0001 *vs* Model group (Normal, *n*=8; Model, *n*=38).


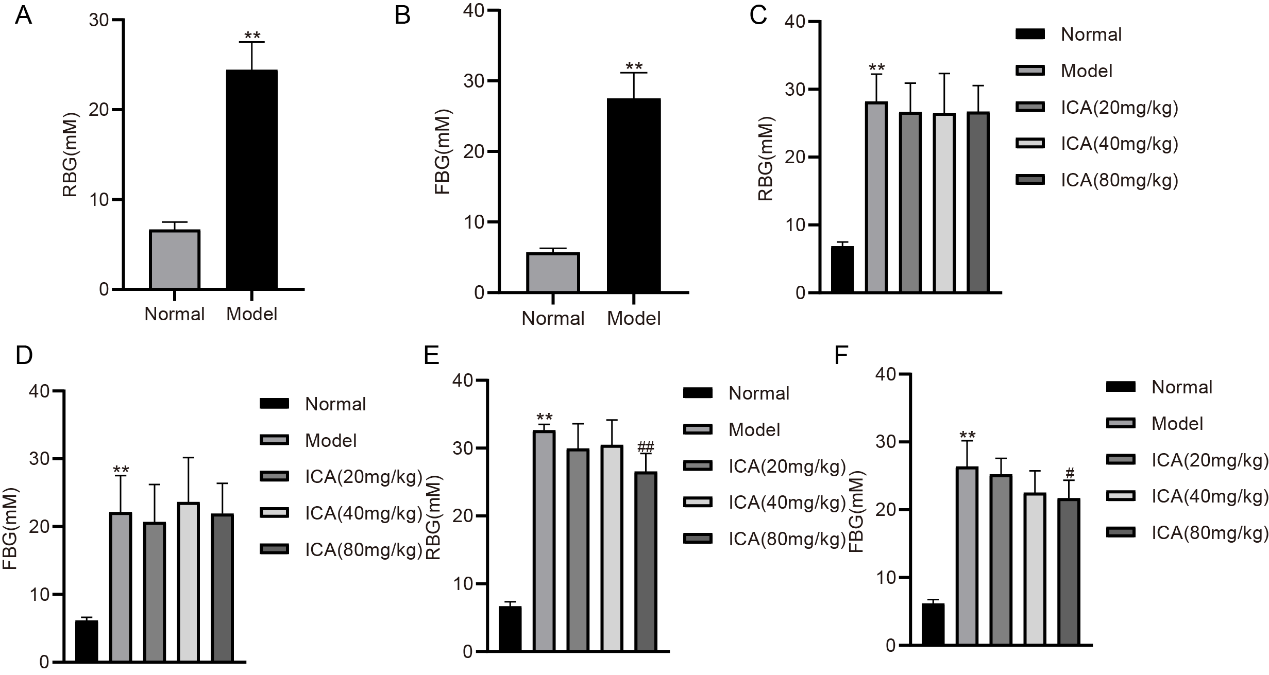


Figure S3 (A) Random blood glucose (RBG) after 72 h of STZ injection (Normal, *n*=8; Model, *n*=40). (B) Fasting blood glucose (FBG) after 1 week of STZ injection (Normal, *n*=8; Model, *n*=38). (C-D) The levels of RBG and FBG after 4 weeks of STZ injection. (E-F) RBG and FBG after 12 weeks of ICA administration. Data are expressed as mean ± SD. ***P*<0.01 *vs* Normal group, *^#^P*<0.05, ^##^*P*<0.01 *vs* Model group.


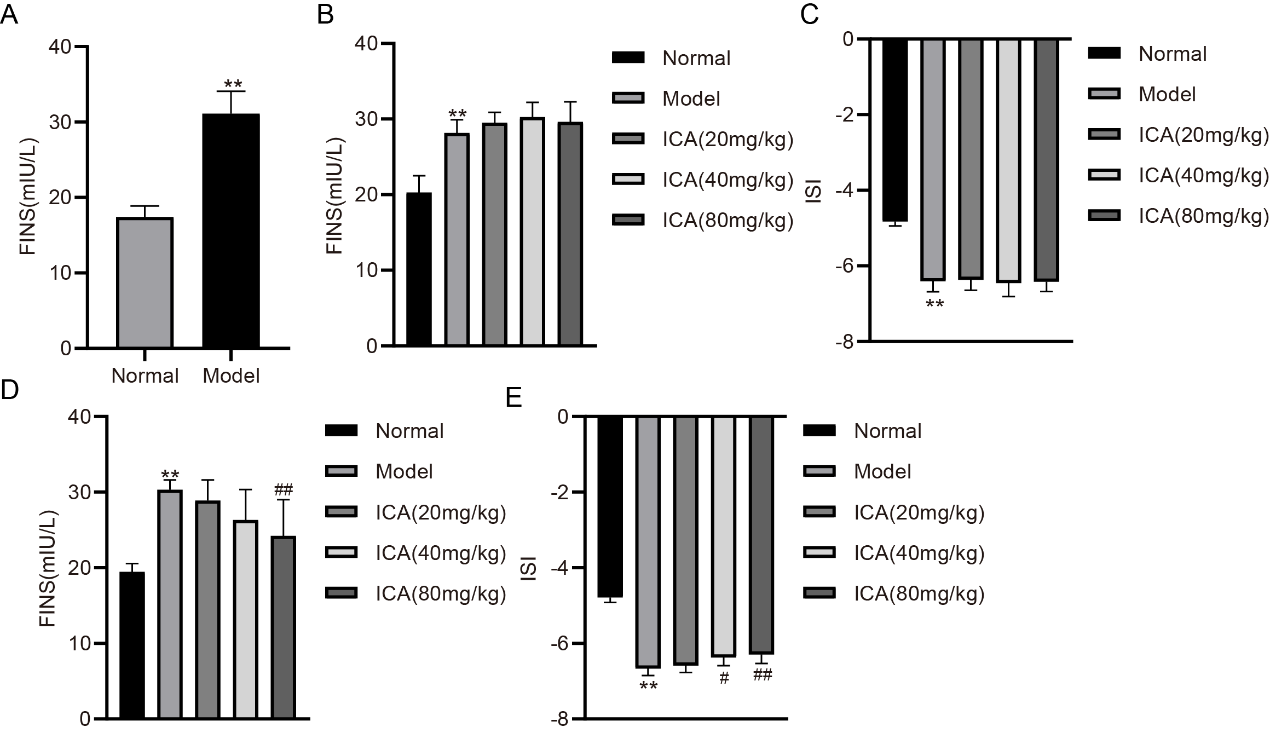


Figure S4 (A) Fasting insulin (FINS) after 4 weeks of high fat and high sugar diet (Normal, *n*=8; Model, *n*=42). (B-C) FINS and insulin sensitivity index (ISI) after 4 weeks of STZ injection. (D-E) FINS and ISI after 12 weeks of ICA administration. ISI= (Ln (1/ (fasting insulin × fasting blood glucose))). Data are expressed as mean ± SD. ***P*<0.01 *vs* Normal group, *^#^P*<0.05, ^##^*P*<0.01 *vs* Model group.


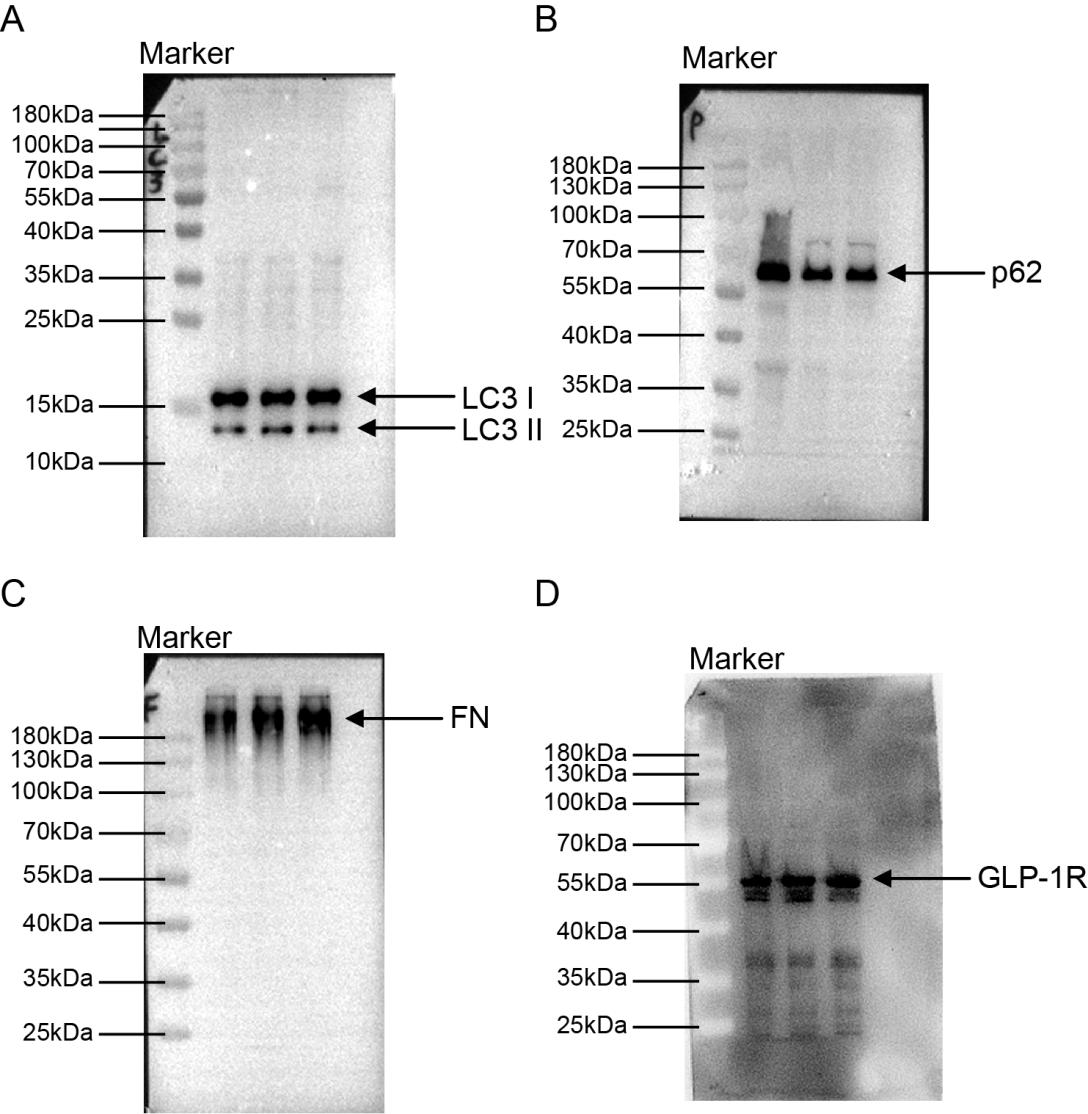


Figure S5 The verification of antibody specificity.
